# The sub-MIC selective window decreases along the digestive tract: determination of the minimal selective concentration of oxytetracycline in sterilised intestinal contents

**DOI:** 10.3389/fmicb.2024.1377159

**Published:** 2024-06-14

**Authors:** Pedro Henrique Imazaki, Bertille Voisin, Nathalie Arpaillange, Béatrice B. Roques, Emilie Dordet-Frisoni, Véronique Dupouy, Aude A. Ferran, Alain Bousquet-Mélou, Delphine Bibbal

**Affiliations:** INTHERES, University of Toulouse, INRAE, ENVT, Toulouse, France

**Keywords:** antibiotic resistance, drug binding, gut, low concentration, minimal selective concentration, risk assessment, sub-inhibitory concentration, tetracycline

## Abstract

**Introduction:**

The administration of antibiotics can expose the digestive microbiota of humans and animals to sub-inhibitory concentrations, potentially favouring the selection of resistant bacteria. The minimal selective concentration (MSC) is a key indicator to understand this process. The MSC is defined as the lowest concentration of an antibiotic that promotes the growth of a resistant strain over a susceptible isogenic strain. It represents the lower limit of the sub-minimal inhibitory concentration (MIC) selective window, where resistant mutants can be selected. Previous studies focused on determining the MSC under standard culture conditions, whereas our research aimed to determine the MSC in a model that approximates *in vivo* conditions.

**Methods:**

We investigated the MSC of oxytetracycline (OTC) in Mueller-Hinton broth (MHB) and sterilised intestinal contents (*SIC*) from the jejunum, caecum and rectum (faeces) of pigs, using two isogenic strains of *Escherichia coli* (one susceptible and one resistant to OTC). Additionally, the MIC of OTC against the susceptible strain was determined to assess the upper limit of the sub-MIC selective window.

**Results:**

Our study took a novel approach, and the results indicated that MIC and MSC values were lower in MHB than in *SIC*. In the latter, these values varied depending on the intestinal segment, with distal compartments exhibiting higher MIC and MSC values. Moreover, the sub-MIC selective window of OTC in SIC narrowed from the jejunum to the rectum, with a significantly closer MSC to MIC in faecal *SIC*.

**Discussion:**

The results suggest that OTC binds to digestive contents, reducing the fraction of free OTC. However, binding alone does not fully explain our results, and interactions between bacteria and intestinal contents may play a role. Furthermore, our findings provide initial estimates of low concentrations facilitating resistance selection in the gut. Finally, this research enhances the understanding of antimicrobial resistance selection, emphasising the intricate interplay between antibiotics and intestinal content composition in assessing the risk of resistance development in the gut.

## 1 Introduction

Antibiotic resistance represents one of our most urgent global health challenges. The primary goal of administering antibiotics to combat bacterial infections is to achieve the highest possible active drug concentration in the infectious site without inducing toxicity to the patient. This optimises cure rates whilst curbing the emergence of *de novo* resistance amongst pathogenic bacteria within the host. However, the administration of antibiotics leads to concentration gradients within the body, resulting in non-targeted bacteria, including those in the gut microbiota, being exposed to low antibiotic concentrations. Studies have shown that such low concentrations of antibiotics could promote the selection of antibiotic-resistant bacteria (Andersson and Hughes, 2014). Thus, potentially resistant bacteria selected in the digestive tract of treated humans and production animals may enter sewage water, slurry and manure, providing conditions that facilitate the spreading of resistance genes in the environment. Therefore, it is essential to determine the minimum antibiotic concentrations potentially responsible for selecting antibiotic-resistant bacteria *in vivo*.

In the past decade, the concept of minimal selective concentration (MSC) has gained importance in the field of antibiotic resistance, allowing for assessing resistance selection at low concentrations. The MSC refers to the lowest concentration of an antibiotic that confers a competitive advantage to a resistant strain over a susceptible isogenic strain (Gullberg et al., 2011). In this manner, exposure of bacteria to antibiotic concentrations within the range of the MSC and the minimal inhibitory concentration (MIC) against the susceptible bacteria (MIC_s_) provides a critical window (sub-MIC selective window) in which resistant bacteria are advantaged whilst still allowing susceptible strains to grow. This phenomenon not only selects and enriches resistant strains but also enhances genetic processes such as horizontal gene transfer, recombination and mutagenesis (Andersson and Hughes, 2014). Furthermore, exposure of bacteria to low concentrations of antibiotics can promote the emergence of resistance through a stepwise accumulation of low-fitness-cost resistance mechanisms, each with minor individual effects (Wistrand-Yuen et al., 2018; Sandegren, 2019). Thus, resistant bacteria with high fitness are selected at low concentrations, which increases the probability of persistence and spread of resistant strains.

Tetracyclines constitute a class of broad-spectrum antibiotics used in both human and veterinary medicine (European Centre for Disease Prevention and Control, 2022; European Medicines Agency, 2022). These antibiotics are primarily administered orally and display highly variable absorption extents, ranging from 25 to 30% for chlortetracycline to over 80% for doxycycline and minocycline. Additionally, certain tetracyclines, including oxytetracycline (OTC), can be eliminated unchanged through biliary or intestinal secretion followed by faecal excretion (Agwuh and MacGowan, 2006). In the gut lumen, the intestinal contents are variable and complex matrices in which antibiotics can bind to various components, leading to a reduction in the concentration of free and active antibiotics along the digestive tract (Vallé et al., 2021). As a result, oral (and even parenteral) administration of antibiotics can expose the intestinal microbiota to sub-inhibitory antibiotic concentrations, either during the treatment as a consequence of incomplete absorption, biliary/intestinal secretion and interactions of the drug with the digestive matrix or after the end of the treatment during the terminal phase of the elimination process.

To date, investigations on the MSC for bacterial species have been conducted in sterile culture broth (Gullberg et al., 2011), culture broth enriched with a pig faecal microbial community (Klümper et al., 2019) and biofilms (Hjort et al., 2022). However, to the best of our knowledge, no experimental study has examined the impact of the intestinal content matrix on the MSC. As reviewed by Murray et al. (2021), several methodological approaches exist to determine the effect of low concentrations of antibiotics. Amongst the diverse approaches, the methodology from Gullberg et al. (2011) presents the advantage of being easily adjustable, permitting it to mimic different environmental conditions. Hence, the present research aimed to establish the MSC of OTC using an adapted protocol based on Gullberg et al. (2011) mimicking conditions encountered in the digestive tract. Specifically, this investigation focused on two under-explored aspects. Firstly, it examined how OTC and bacteria interact with the constituents of the intestinal contents in the context of MSC determination. Secondly, it aimed to assess the extent of the sub-MIC selective window along the digestive tract.

## 2 Materials and methods

### 2.1 Bacterial strains and growth conditions

Two isogenic strains of *Escherichia coli*, DA34574 and DA34433, were used in this study. These strains were obtained from the Department of Biochemistry and Medical Microbiology at Uppsala University, Sweden, and both are derivatives of *E. coli* MG1655. Strain DA34574 was susceptible to tetracyclines, whilst strain DA34433 possessed the non-conjugative plasmid pCA24N(gfp-)-tet(G), which conferred resistance to tetracyclines through the expression of efflux pumps (Nicoloff and Andersson, 2016). Cultures of these strains were carried out in 5 mL of Muller-Hinton broth (MHB) at 37°C whilst shaking (180 rpm). For strain DA34433, 6.0 μg/mL of OTC was added to the culture media to preserve the plasmid carrying the antibiotic resistance gene.

### 2.2 Preparation of sterilised intestinal contents (*SIC*)

In order to take into account individual variation, four White Large pigs aged between 55 and 56 days and weighing between 14.7 and 21.45 kg at the time of the study were used. They had access to water and feed (flour-based growth food [18.0% protein, 7.0% fat, 4.4% cellulose and 3.8% ash], PS2, Solevial, Villefranche de Rouergue, France) *ad libitum*.

After euthanasia, the contents of the jejunum, caecum and rectum were removed and collected in tubes. These tubes were placed on ice and stored at −20°C. The intestinal contents stored at −20°C were thawed at room temperature and pooled. The contents were weighed and placed in a bag with a filter. Mueller-Hinton broth (MHB) was added to achieve an intestinal content:MHB ratio of 1:5 w/v. The resulting filtered suspensions were mixed using a homogeniser blender for 90 s. Then, they were sterilised by autoclaving at 121°C for 20 min to obtain sterilised intestinal contents (*SIC*) from the jejunum, caecum and rectum (faecal content). The obtained *SIC* were stored at −20°C before use.

The French Ministry of Research authorised the experimental protocol (#23387_2019122010002751), and the study was conducted in accordance with local legislation and institutional requirements.

### 2.3 Evaluation of the bacterial growth

Overnight cultures of strains DA34574 and DA34433 were handled individually. First, each culture was centrifuged, and the supernatant was discarded. Then, the resulting pellet for each strain was rinsed with phosphate-buffered saline (PBS), centrifuged once more, and resuspended in an equal volume of MHB. These suspensions were subsequently diluted to achieve a final concentration of 10^3^ CFU/mL in a fresh medium (MHB or *SIC*) without antibiotics and incubated at 37°C for 24 h. During the incubation, aliquots were collected hourly for 8 h, with each aliquot being plated in triplicate on Mueller-Hinton agar (MHA). An additional aliquot was taken after 24 h and plated in triplicate on MHA. The plates were then incubated overnight, and the colonies were counted the following day. This experiment was conducted in triplicate, and the growth kinetics were represented as a curve showing colony-forming units per millilitre over time in hours.

### 2.4 Determination of the minimal inhibitory concentration (MIC)

The MIC determination was conducted using the microdilution method. Bacterial strains were exposed to different ranges of antibiotic concentrations in MHB or *SIC*, with a two-fold dilution factor between each concentration. For strain DA34574 (susceptible), which had a previously reported MIC of 0.38 μg/mL (Nicoloff and Andersson, 2016), an OTC range of 0.125 to 64 μg/mL was tested. Strain DA34433 (resistant), with an indicated MIC of 64 μg/mL (Nicoloff and Andersson, 2016), was subjected to an OTC range of 1 to 512 μg/mL. Bacterial suspensions of both strains were prepared in MHB, starting from colonies isolated on MHA, and diluted to achieve an approximate concentration of 10^5^ CFU/mL in each well of the microplate. Subsequently, the plates were inoculated and then incubated overnight at 37°C.

After incubation, the bacterial suspensions from each well were serially diluted up to a dilution factor 10^−6^. Next, 10 μL of each dilution was plated in triplicate on charcoal trypticase soy agar. The plates were then incubated overnight, and colony counts were performed the following day. The MIC value corresponded to the highest OTC concentration at which at least 10^5^ CFU/mL were observed, indicating a bacteriostatic effect.

### 2.5 Competition experiments and determination of the minimal selective concentration (MSC)

This experiment aimed to determine the minimum antibiotic concentration that confers a selective advantage to a resistant strain compared to a susceptible isogenic strain. Co-cultures of strains DA34574 (susceptible) and DA34433 (resistant) were conducted in MHB and *SIC* at varying concentrations of OTC, all being lower than the MIC of the susceptible strain DA34574.

An adapted protocol based on Gullberg et al. (2011) was developed. Initially, overnight cultures of both strains were centrifuged, the supernatant was discarded, and the pellet was resuspended in PBS. The pellet of the strain DA34433 was rinsed with PBS to remove any remaining antibiotics. Both strains were co-cultured with an initial bacterial load of 10^5^ CFU/mL in either MHB or *SIC* at different OTC concentrations.

Following co-culturing and after 24 and 48 h of incubation, the total bacterial counts (comprising both strains) were determined on MHA, whilst the counts of the resistant strain (DA34433) were determined on MHA supplemented with OTC at 6 μg/mL. The number of susceptible bacteria was calculated by subtracting the counts of resistant bacteria from the total counts. The ratio of resistant to susceptible bacteria was monitored over time, and the selection coefficient was determined using the equation:


$$s=\frac{\ln\left(\frac{R_{\left(t\right)}}{R_{\left(0\right)}}\right)}{t}$$

Where *s* represents the selection coefficient, *R_(t)_* indicates the resistant to susceptible bacteria ratio after 48 h, *R_(0)_* is the initial ratio at the beginning of the co-culture and *t* is the co-culture time (48 h). The calculated selection coefficients were then plotted as a function of the OTC concentration in each culture. The MSC was determined through interpolation using a polynomial model representing the OTC concentration at which the selection coefficient equals 0.

### 2.6 Statistical analysis

Bacterial growth data were analysed using three-way analysis of variance (RStudio Team, 2020). The effects of strain (DA34574 and DA34433), medium (MHB and jejunum, caecum and faecal *SIC*) and time points (0, 1, 2, 3, 4, 5, 6, 7, 8 and 24 h) and all their interactions on bacterial counts were calculated.

## 3 Results

### 3.1 Evaluation of the bacterial growth

**Figure 1:**
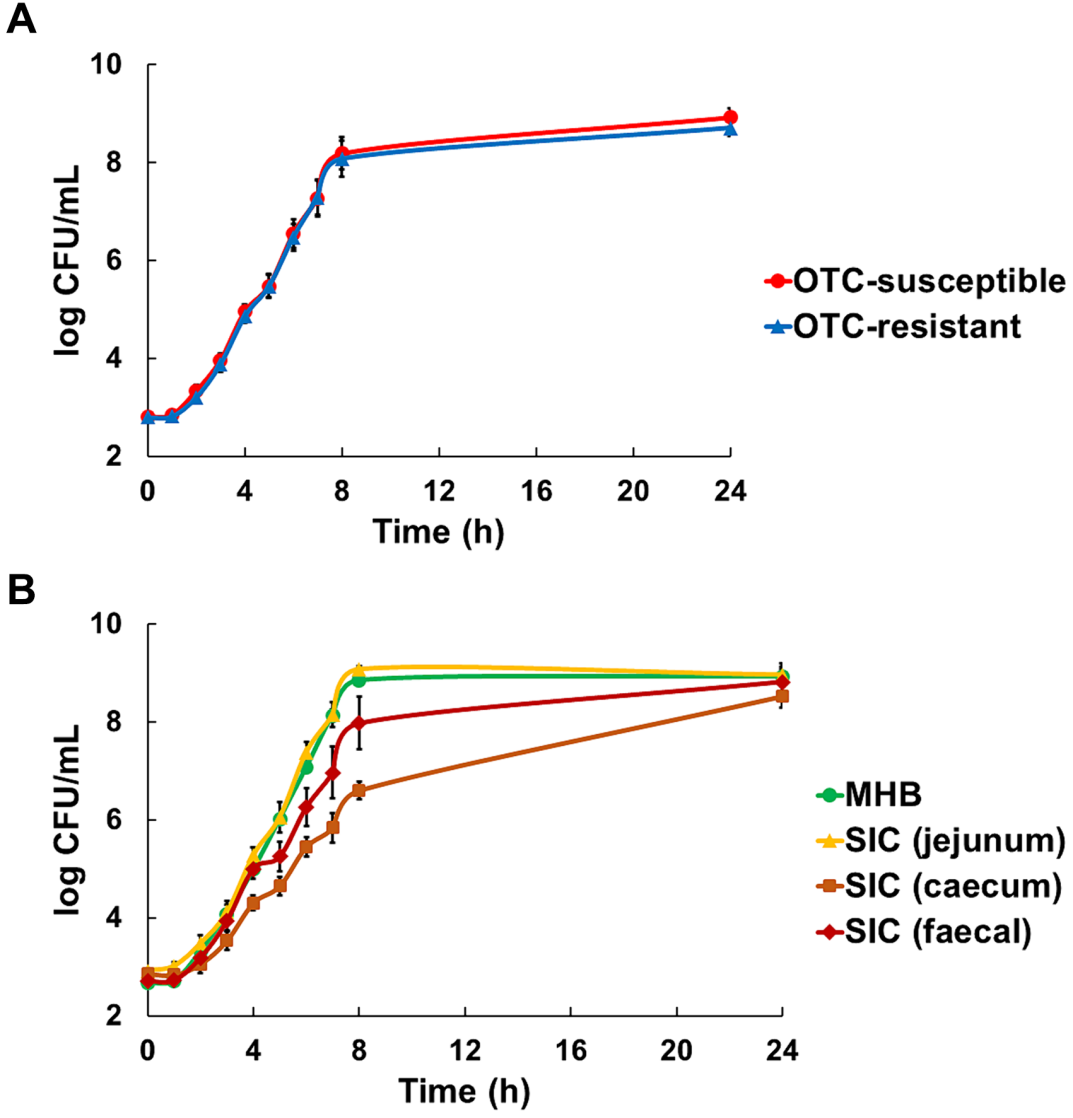
Evaluation of the growth of oxytetracycline (OTC)-susceptible (DA34574) and OTC-resistant (DA34433) isogenic *Escherichia coli* strains in Mueller-Hinton broth (MHB) and jejunum, caecum and faecal sterilised intestinal contents (*SIC*) of pigs. Panel **(A)** shows the strain effect [OTC-susceptible (●) and OTC-resistant (▲)] in MHB and jejunum, caecum and faecal *SIC* (*n* = 12). Panel **(B)** shows the medium effect [MHB (●), jejunum *SIC* (▲), caecum *SIC* (■) and faecal *SIC* (◆)] for both OTC-susceptible and resistant strains (*n* = 6). Bars represent standard error.

In all media (MHB and *SIC*), the susceptible (DA34574) and resistant (DA34433) strains showed identical growth kinetics, confirming the absence of strain-related effects and ensuring that subsequent results were not influenced by variations in growth rates between the two strains (Figure 1A). However, significant variations were observed between the different media conditions (*p* < 0.05). Specifically, slower growth rates were observed in caecum *SIC* (with bacterial counts ranging from 6.6 ± 0.2 to 8.5 ± 0.2 log_10_ CFU/mL after 8 and 24 h of culture, respectively), whilst faster growth rates were observed in MHB and jejunum *SIC* (where bacterial already reached 8.9 ± 0.1 and 9.1 ± 0.1 after 8 h of culture in MHB and jejunum *SIC*, respectively) (Figure 1B).

### 3.2 Minimal inhibitory concentration (MIC) of oxytetracycline (OTC)

**Figure 2:**
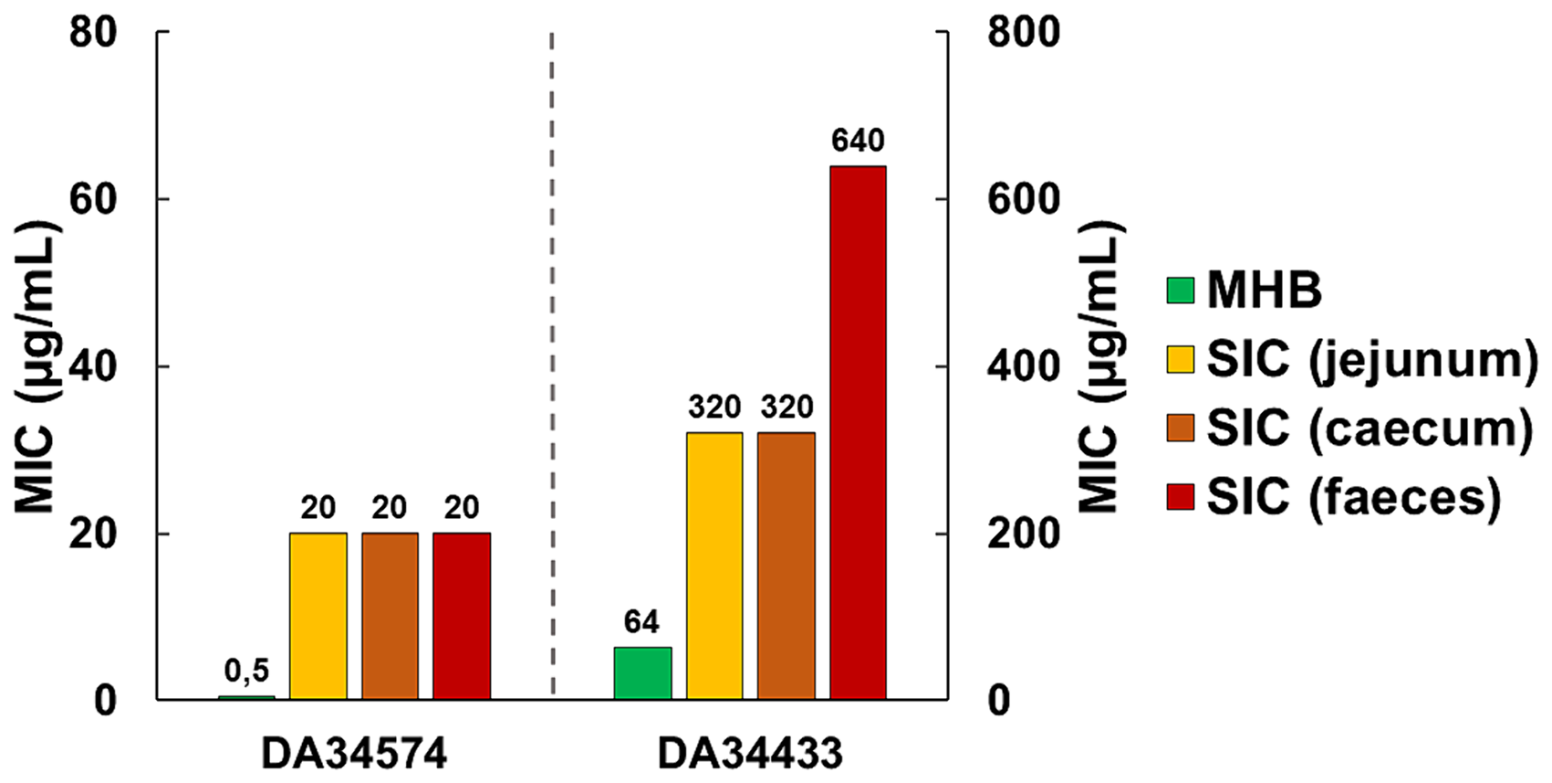
Minimal inhibitory concentration (MIC) of oxytetracycline (OTC) against OTC-susceptible (DA34574) and OTC-resistant (DA34433) isogenic *Escherichia coli* strains in Mueller-Hinton broth (MHB) and jejunum, caecum and faecal sterilised intestinal contents (*SIC*) of pigs.

In MHB, the OTC-susceptible strain DA34574 displayed a MIC value of 0.5 μg/mL, whilst the OTC-resistant strain DA34433 exhibited a higher MIC value of 64 μg/mL. In intestinal contents, the OTC-susceptible strain showed a consistent MIC of 20 μg/mL across jejunum, caecum and faecal *SIC*. In contrast, the OTC-resistant strain exhibited varying MIC values depending on the intestinal content, from 320 μg/mL in jejunum and caecum *SIC* to 640 μg/mL in faecal *SIC*. These results indicate a variation in the MIC depending on the specific intestinal content. Overall, the MIC was 40-fold higher in *SIC* compared to MHB for the OTC-susceptible strain and 5- to 10-fold higher in *SIC* compared to MHB for the OTC-resistant strain (Figure 2). In summary, these results show a different impact of the intestinal contents on the MIC of OTC according to the strains.

### 3.3 Minimal selective concentration (MSC) of oxytetracycline (OTC)

**Figure 3:**
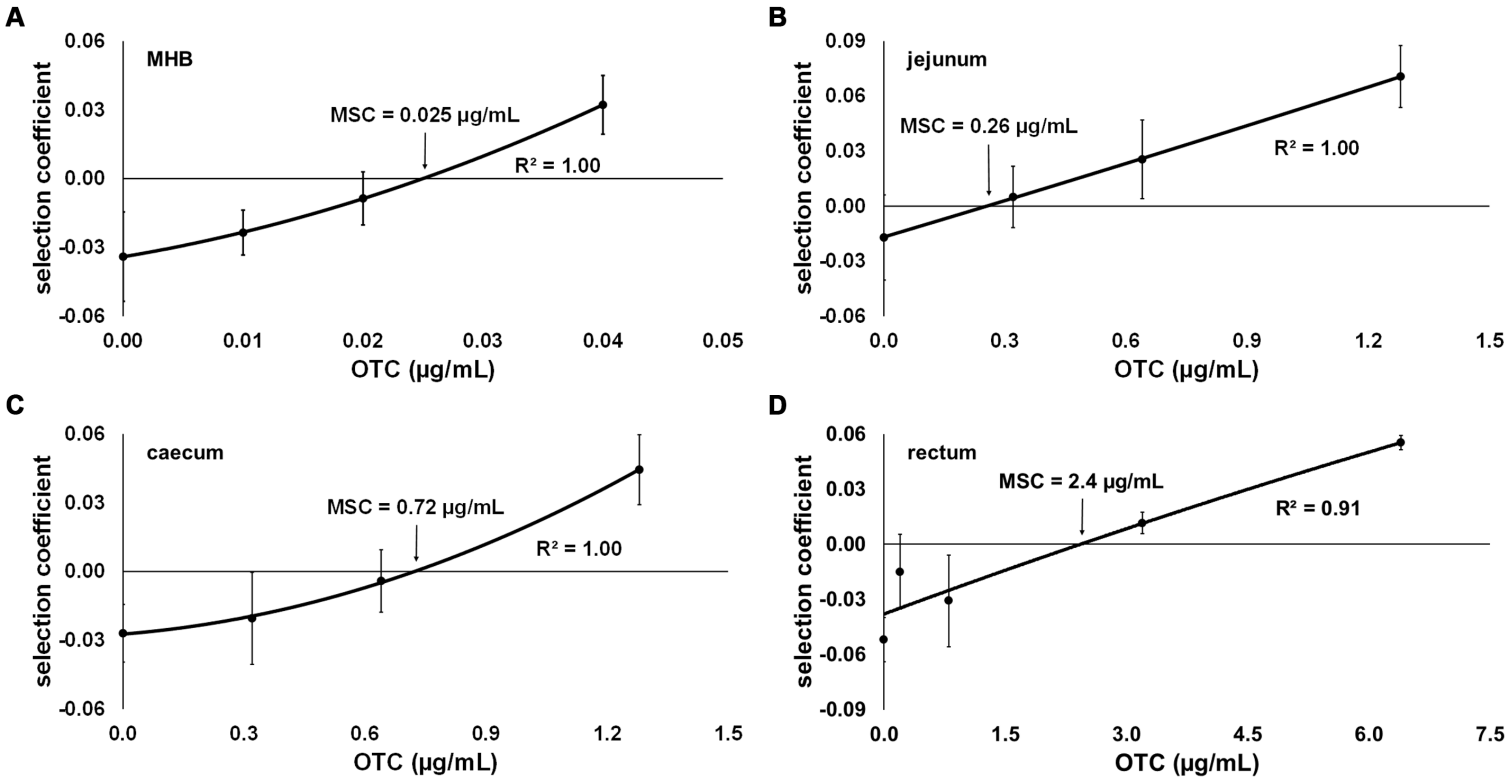
Selection coefficient of an oxytetracycline (OTC)-resistant strain (DA34433) against an OTC-susceptible isogenic *Escherichia coli* strain (DA34574) as a function of OTC concentration in **(A)** Mueller-Hinton broth (MHB) and sterilised intestinal content of the **(B)** jejunum, **(C)** caecum and **(D)** rectum from pigs. The OTC concentration at which the selection coefficient is 0 represents the minimal selective concentration (MSC). Bars represent standard error.

When determining the MSC of OTC in MHB, concentrations of 0, 0.01, 0.02 and 0.04 μg/mL of OTC were selected based on preliminary co-culture tests, which indicated that the selection coefficient approaches 0 within this concentration range. The selection coefficient was calculated over the 48-h co-culture period and yielded the following values for the tested OTC concentrations: −0.034 (0 μg/mL), −0.024 (0.01 μg/mL), −0.009 (0.02 μg/mL) and 0.032 (0.04 μg/mL). These findings suggest that the OTC-resistant strain had a competitive advantage at OTC concentrations above 0.02, as indicated by the selection coefficient becoming positive between 0.02 and 0.04 μg/mL. After plotting the selection coefficient against OTC concentration and interpolation to determine the OTC concentration at which the selection coefficient equals 0, the calculated MSC for OTC in MHB was 0.025 μg/mL (Figure 3A).

Assuming that antibiotics bind to the digestive matrices’ contents, and based on preliminary tests, higher OTC concentrations were employed in the co-culture assays using *SIC*: 0, 0.32, 0.64 and 1.28 μg/mL for jejunum and caecum *SIC* and 0, 0.8, 3.2 and 6.4 μg/mL for faecal *SIC*. As anticipated, an increase in the MSC value for OTC was observed depending on the content of each studied digestive compartment. Specifically, the more distal the compartment from which the content was used in the medium, the more the MSC value increased compared to MHB. The MSC values for OTC in jejunum, caecum and faecal *SIC* were 0.26, 0.72, and 2.5 μg/mL, respectively (Figures 3B–D).

## 4 Discussion

The present study aimed to contribute to the assessment of the risk of selecting antimicrobial resistance at very low antibiotic concentrations that can occur within specific body compartments during antibiotic treatment. More precisely, we evaluated the impact of the intestinal environment on the values of MSC, the determination of which is a prerequisite for implementing strategies to decrease antibiotic concentrations and avoid prolonged exposure of commensal bacteria to sub-MIC levels of antibiotics (Gullberg et al., 2011). Whilst previous research on the selection of resistance at low concentrations of antibiotics in the gut has focused on the role of a natural microbial community on the MSC (Klümper et al., 2019), we were interested in a parameter not studied until now: the digestive matrix. For this purpose, we used *SIC* from different segments of the pig digestive tract to determine the MSC of OCT for *E. coli* strains *in vitro*. The *SIC* were used to evaluate the impact of the physicochemical characteristics of intestinal fluids and contents on the determination of the MSC, as well as the consequences on the sub-MIC selective window along the digestive tract.

The first comparative bacterial growth experiments indicated that the OTC-susceptible and OTC-resistant isogenic *E. coli* strains grew similarly in each medium, namely MHB and jejunum, caecum and faecal *SIC*. These observations confirmed the absence of a potential cause of bias, such as differential growth rates, during the co-culture assays. However, growth rate variations between the strains were observed across the media, with faster growth in MHB and jejunum *SIC* compared to caecum and faecal *SIC*. Several hypotheses can be proposed to explain the observed growth variations. First, it is well documented that the microbiota composition and concentration vary along different digestive tract segments (Sundin et al., 2017), and these variations, along with the digestive metabolism of animals, contribute to the differential breakdown of the constituents of the digestive matrix in each segment (Tan et al., 2018). Consequently, the nutrient composition within the different segments could differ, potentially influencing microbial growth. Additionally, the physicochemical properties of the contents within each segment, such as viscosity (McDonald et al., 2001) and fibre content (Makki et al., 2018), can influence microbial growth. Although not directly tested in our study, it is plausible that the combination of nutrient content variations and the physicochemical nature of the contents within different intestinal segments shaped microbial growth patterns.

The determination of the MIC in MHB and *SIC* revealed an increase in MIC values when determined in *SIC*. This increase in the intestinal contents was expected, as previous studies by Ferran et al. (2013) and Vallé et al. (2021) have demonstrated a decrease in antibiotic activity in the presence of the intestinal matrix. Moreover, Ahn et al. (2018) established that nearly 60% of tetracycline binds to human faeces, as determined through chemical and microbiological assays. Consequently, the fraction of OTC that remains unbound and thus potentially active is lower in *SIC*, necessitating a higher overall concentration of the antibiotic in the intestinal contents to achieve the same bacteriostatic effect as in MHB. Although the binding of OTC to the matrix is hypothetical, and the determination of MIC does not directly inform about the free fraction of the antibiotic, this hypothesis is pertinent in interpreting our results. To illustrate, the 10-fold increase in the MIC for the OTC-resistant strain in faecal *SIC* compared to MHB and the 2-fold increase compared to jejunum and caecum *SIC* can be attributed to the change in matrix. This difference likely results in stronger binding of OTC to the components of distal intestinal segments compared to proximal segments, as observed by Ferran et al. (2013) and Vallé et al. (2021).

Furthermore, we observed a difference in the extent of the increase in MIC values between the OTC-susceptible strain and the OTC-resistant strain during the transition to the intestinal contents. Interestingly, the MIC for the OTC-susceptible strain increased by a factor of 40 across the three different digestive contents when compared to MHB. In contrast, the MIC for the OTC-resistant strain only increased by a factor of five when transitioning from MHB to jejunum or caecum *SIC* and by a factor of 10 in faecal *SIC*. A possible explanation for this result is the saturation of the matrix when determining the MIC at high concentrations. If OTC binds to the matrix, it can be assumed that the interaction continues until saturation occurs. When cultured at high antibiotic concentrations, the matrix becomes saturated more quickly, resulting in a higher proportion of free (potentially active) OTC. Therefore, in determining the MIC for the OTC-resistant strain in faecal *SIC*, it is possible that the matrix reached saturation, leading to a lower ratio of MIC in faecal *SIC* to MIC in MHB for the OTC-resistant strain (10) than observed for the OTC-susceptible strain (40). Finally, we cannot exclude the possibility that although the viable microbiota was eliminated in our study by heat sterilisation, dead bacterial cells could have adsorbed antibiotics, potentially impacting the concentration available for selection, as confirmed by Podlesek et al. (2016).

The determination of the MSC in MHB and *SIC* revealed an increase in MSC values in *SIC* compared to MHB. This finding aligns with the observations made during the determination of the MIC, suggesting a higher interaction between OTC and the intestinal contents from distal compartments of the digestive tract. However, the variations in MIC and MSC were not proportional across the different intestinal segments. Given that the MSC values are lower than the OTC concentration range where saturation of the intestinal matrix is plausible (as observed with the MIC values), another hypothesis to consider involves the interaction between bacteria and the intestinal contents. This interaction could create an environmental niche, providing some protection against the antibiotic. This hypothesis is supported by a study by Vallé et al. (2021), which demonstrated with another tetracycline, minocycline, that a combination of factors decreasing available antibiotic concentrations and reducing bacterial susceptibility to unbound antibiotic could account for the effects observed in *SIC*.

**Figure 4:**
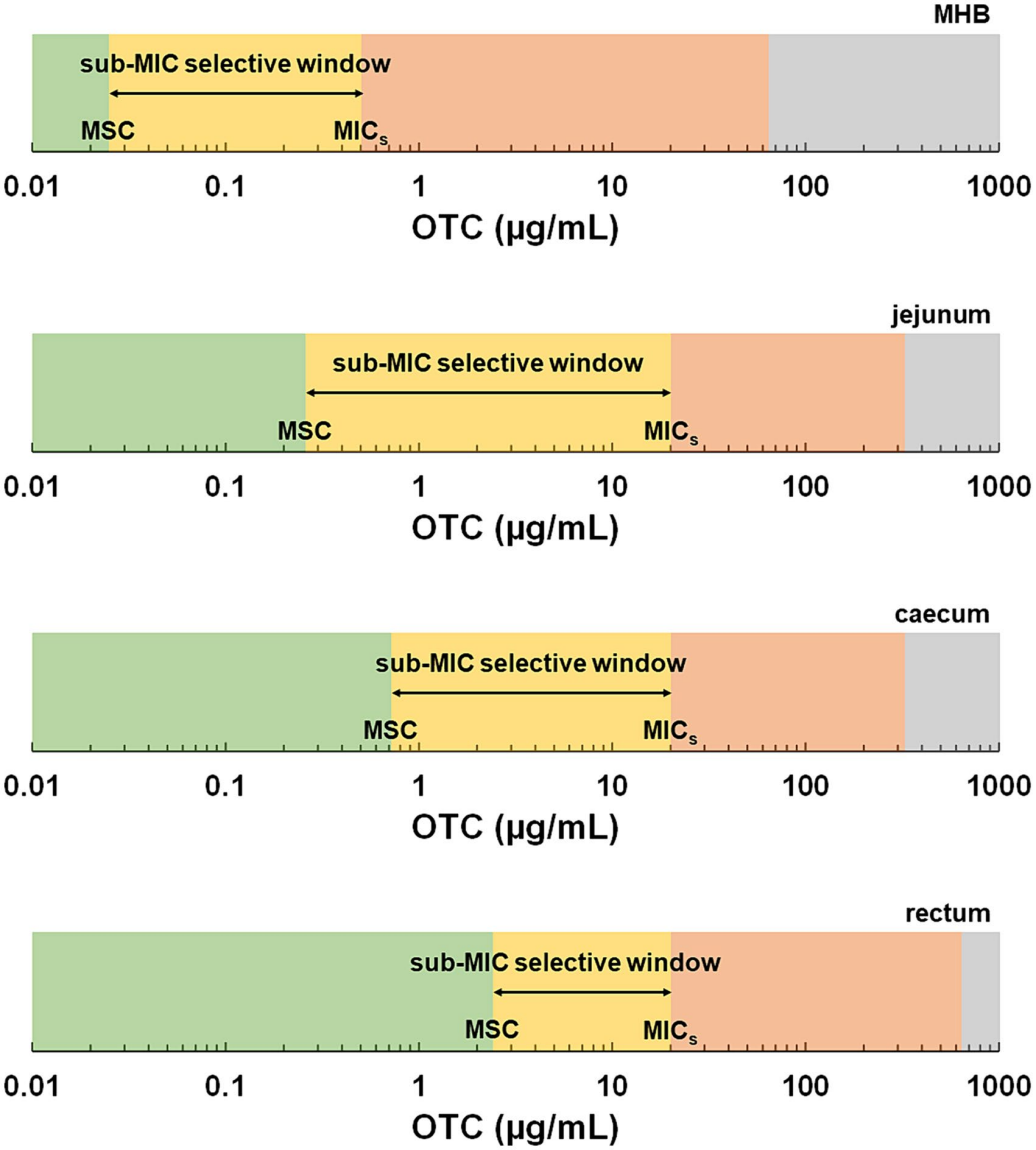
Schematic representation of selective windows based on the minimal selective concentrations (MSC) and minimal inhibitory concentrations (MIC) determined in this study as a function of the medium. The green zone represents concentrations below the MSC. The yellow zone represents the sub-MIC selective window, the range of concentrations between the MSC and the minimal inhibitory concentration of the susceptible strain (MIC_s_). The red zone represents the traditional selective window, the range of concentrations above the MIC_s_ and below the MIC of the resistant strain, where only the resistant strain can grow. The grey area represents the zone above the MIC of the resistant strain.

The MSC and the MIC for the OTC-susceptible strain define a range of concentrations where resistance selection can occur at very low levels. This sub-MIC selective window not only facilitates the horizontal transfer of resistance genes between resistant and susceptible strains but also promotes the emergence of *de novo* mutations (Hughes and Andersson, 2012). Indeed, the selection pressure imposed by low concentrations of antibiotics leads to an increased mutation frequency in susceptible bacteria, enabling their survival. However, these mutations often involve the development of resistance mechanisms with low biological costs, allowing the favoured growth of antibiotic-resistant strains over susceptible strains (Sandegren, 2014). Previous studies investigating the MSC of antibiotics in culture broth and aquatic environments have demonstrated the presence of sub-MIC selective windows encompassing concentrations several tenths-fold below MIC values (Gullberg et al., 2011; Kraupner et al., 2020). In the context of the highly abundant and diverse microbiota within the digestive tract, such sub-MIC selective windows would pose significant concerns. In our study, the MSC in jejunum *SIC* was about 80 times lower than the MIC for the OTC-susceptible strain, whilst it was 28 times lower in caecum *SIC*. However, notably, the MSC in faecal *SIC*, representing the compartment with a significantly more abundant digestive microbiota, was only eight times lower. Consequently, we observed a reduction in the sub-MIC selective window along the digestive tract (Figure 4), providing novel insights into the risk assessment of antimicrobial resistance selection in the gut. However, the lack of previous studies on the MSC in the presence of the digestive matrix prevented a direct comparison of the results of this study.

Besides the interactions between the antibiotic, target bacteria and digestive matrix, the digestive microbiota could also narrow the range of sub-inhibitory concentrations favouring resistance selection. This hypothesis is mainly supported by Klümper et al.’s (2019) study, which explored the impact of a microbial community on the MSC. This study found that resistance selection was less pronounced in the presence of the digestive microbiota, attributable to the complex community’s protective effect against antibiotic activity. Consequently, the actual MSC in the digestive tract is likely higher than estimated in our study, where resident microbiota was removed by sterilisation. Thus, employing a dynamic system of bioreactors would be beneficial to reflect both the effects of digestive matrices and the intestinal microbiota and to determine the MSC of OTC under conditions closer to *in vivo*. Such a system would allow for the simulation of various compartments of the digestive tract, controlling physiological aspects like pH, as well as the microflora present in each reactor, as suggested by Dufourny et al. (2019).

It should be highlighted that the physicochemical characteristics of intestinal fluids can vary dramatically between species, influenced by their feeding behaviour and associated digestive physiology, as well as within a species depending on the type of feed, for example. These factors might significantly influence the values of MSC of an antibiotic both between species and within individuals of the same species. Such variability could be seen as a limitation when determining MSC in biological contents, necessitating its determination in specific environmental conditions, as opposed to controlled broths. Whilst the purpose of the present study was not to investigate the variability of the physicochemical characteristics of intestinal fluids, it underscores the importance of considering this factor in the determination of MSC. Moreover, we tested only one antibiotic in this study. The diverse chemical nature of antibiotics may also play a role in the extent of binding, and the fitness cost associated with resistance mechanisms for different antibiotics may influence the minimal antibiotic concentration at which resistant bacteria outcompete susceptible bacteria.

In perspective, further investigations are warranted to fully understand the implications of sub-inhibitory concentrations of antibiotics on the selection of antibiotic resistance. It is essential to verify whether the active concentrations of OTC in the digestive tract after treatment of infectious diseases in humans or animals align with the concentration window identified in this study. Quantifying the total amount of OTC in the digestive contents and estimating the potentially active fraction (free fraction) in different contents can provide additional elements to assess the risk of antimicrobial resistance selection. Additionally, exploring the influence of the intestinal microbiota on the MSC would be beneficial. The presence of a microbial community is expected to impact the MIC and MSC values, potentially altering resistance selection. Moreover, conducting molecular studies would enable the investigation of the transfer of resistance genes and the selection of associated resistance mechanisms at low concentrations of antibiotics.

In summary, this study provides valuable insights into the emergence of antimicrobial resistance in the gut environment. By analysing the MSC of OTC in different sterilised intestinal contents, we identified the window of sub-inhibitory concentrations contributing to resistance selection and the growth advantage of resistant strains over susceptible isogenic strains along the digestive tract. Furthermore, the methodology developed in this research can be applied to other antibiotics, and the findings obtained may have implications for developing improved guidelines for antibiotic administration.

## Data availability statement

The raw data supporting the conclusions of this article will be made available by the authors, without undue reservation.

## Ethics statement

The animal study was approved by French Ministry of Research Experimental protocol #23387_2019122010002751. The study was conducted in accordance with the local legislation and institutional requirements.

## Author contributions

PI: Conceptualisation, Data curation, Formal analysis, Funding acquisition, Methodology, Investigation, Project administration, Validation, Visualisation, Writing – original draft, Writing – review & editing. BV: Conceptualisation, Data curation, Formal analysis, Methodology, Investigation, Project administration, Validation, Visualisation, Writing – original draft. NA: Conceptualisation, Data curation, Investigation, Resources, Writing – original draft. BR: Conceptualisation, Data curation, Methodology, Investigation, Resources, Writing – original draft. ED-F: Conceptualisation, Writing – original draft. VD: Conceptualisation, Investigation, Writing – original draft. AF: Conceptualisation, Methodology, Investigation, Writing – original draft. AB-M: Conceptualisation, Formal analysis, Funding acquisition, Methodology, Supervision, Writing – original draft. DB: Conceptualisation, Investigation, Supervision, Writing – original draft.

## References

[ref1] Agwuh K. N. MacGowan A. (2006). Pharmacokinetics and pharmacodynamics of the tetracyclines including glycylcyclines. J. Antimicrob. Chemother. 58, 256–265. doi: 10.1093/jac/dkl224, PMID: 16816396

[ref2] Ahn Y. Jung J. Y. Veach B. T. Khare S. Gokulan K. Piñeiro S. A.. (2018). *In vitro* test systems to determine tetracycline residue binding to human feces. Regul. Toxicol. Pharmacol. 99, 105–115. doi: 10.1016/j.yrtph.2018.09.013, PMID: 30227174 PMC12423784

[ref3] Andersson D. Hughes D. (2014). Microbiological effects of sublethal levels of antibiotics. Nat. Rev. Microbiol. 12, 465–478. doi: 10.1038/nrmicro3270 24861036

[ref4] Dufourny S. Everaert N. Lebrun S. Douny C. Scippo M. L. Li B.. (2019). Baby-SPIME: a dynamic in vitro piglet model mimicking gut microbiota during the weaning process. J. Microbiol. Methods 167:105735. doi: 10.1016/j.mimet.2019.105735, PMID: 31669849

[ref5] European Centre for Disease Prevention and Control (2022). Antimicrobial consumption in the EU/EEA (ESAC-net): Annual epidemiological report 2021. Stockholm: ECDC.

[ref6] European Medicines Agency (2022). Sales of veterinary antimicrobial agents in 31 European countries in 2021: Twelfth ESVAC report. Luxembourg: Publications Office of the European Union.

[ref8] Ferran A. A. Bibbal D. Pellet T. Laurentie M. Gicquel-Bruneau M. Sanders P.. (2013). Pharmacokinetic/pharmacodynamic assessment of the effects of parenteral administration of a fluoroquinolone on the intestinal microbiota: comparison of bactericidal activity at the gut versus the systemic level in a pig model. Int. J. Antimicrob. Agents 42, 429–435. doi: 10.1016/j.ijantimicag.2013.07.008, PMID: 24021905

[ref9] Gullberg E. Cao S. Berg O. G. Ilbäck C. Sandegren L. Hughes D.. (2011). Selection of resistant bacteria at very low antibiotic concentrations. PLoS Pathog. 7:e1002158. doi: 10.1371/journal.ppat.1002158, PMID: 21811410 PMC3141051

[ref10] Hjort K. Fermér E. Tang P. C. Andersson D. I. (2022). Antibiotic minimal selective concentrations and fitness costs during biofilm and planktonic growth. MBio 13:e0144722. doi: 10.1128/mbio.01447-22, PMID: 35695458 PMC9239065

[ref11] Hughes D. Andersson D. I. (2012). Selection of resistance at lethal and non-lethal antibiotic concentrations. Curr. Opin. Microbiol. 15, 555–560. doi: 10.1016/j.mib.2012.07.005, PMID: 22878455

[ref12] Klümper U. Recker M. Zhang L. Yin X. Zhang T. Buckling A.. (2019). Selection for antimicrobial resistance is reduced when embedded in a natural microbial community. ISME J. 13, 2927–2937. doi: 10.1038/s41396-019-0483-z, PMID: 31384011 PMC6864104

[ref13] Kraupner N. Ebmeyer S. Hutinel M. Fick J. Flach C. F. Larsson D. G. J. (2020). Selective concentrations for trimethoprim resistance in aquatic environments. Environ. Int. 144:106083. doi: 10.1016/j.envint.2020.106083, PMID: 32890888

[ref15] Makki K. Deehan E. C. Walter J. Bäckhed F. (2018). The impact of dietary fiber on gut microbiota in host health and disease. Cell Host Microbe 23, 705–715. doi: 10.1016/j.chom.2018.05.012 29902436

[ref16] McDonald D. Pethick D. Mullan B. Hampson D. (2001). Increasing viscosity of the intestinal contents alters small intestinal structure and intestinal growth, and stimulates proliferation of enterotoxigenic *Escherichia coli* in newly-weaned pigs. Br. J. Nutr. 86, 487–498. doi: 10.1079/BJN2001416, PMID: 11591236

[ref17] Murray A. K. Stanton I. Gaze W. H. Snape J. (2021). Dawning of a new ERA: environmental risk assessment of antibiotics and their potential to select for antimicrobial resistance. Water Res. 200:117233. doi: 10.1016/j.watres.2021.117233, PMID: 34038824

[ref18] Nicoloff H. Andersson D. I. (2016). Indirect resistance to several classes of antibiotics in co-cultures with resistant bacteria expressing antibiotic-modifying or -degrading enzymes. J. Antimicrob. Chemother. 71, 100–110. doi: 10.1093/jac/dkv312 26467993

[ref19] Podlesek Z. Butala M. Šakanović A. Žgur-Bertok D. (2016). Antibiotic induced bacterial lysis provides a reservoir of persisters. Antonie Van Leeuwenhoek 109, 523–528. doi: 10.1007/s10482-016-0657-x, PMID: 26821377

[ref20] RStudio Team (2020). RStudio: Integrated development for R. PBC, Boston, MA: RStudio.

[ref21] Sandegren L. (2014). Selection of antibiotic resistance at very low antibiotic concentrations. Ups. J. Med. Sci. 119, 103–107. doi: 10.3109/03009734.2014.904457 24694026 PMC4034545

[ref22] Sandegren L. (2019). Low sub-minimal inhibitory concentrations of antibiotics generate new types of resistance. Sustain. Chem. Pharm. 11, 46–48. doi: 10.1016/j.scp.2018.12.006

[ref24] Sundin O. H. Mendoza-Ladd A. Zeng M. Diaz-Arévalo D. Morales E. Fagan B. M.. (2017). The human jejunum has an endogenous microbiota that differs from those in the oral cavity and colon. BMC Microbiol. 17:160. doi: 10.1186/s12866-017-1059-6, PMID: 28716079 PMC5513040

[ref25] Tan Z. Wang Y. Yang T. Ao H. Chen S. Xing K.. (2018). Differences in gut microbiota composition in finishing landrace pigs with low and high feed conversion ratios. Antonie Van Leeuwenhoek 111, 1673–1685. doi: 10.1007/s10482-018-1057-1, PMID: 29497869 PMC6097733

[ref27] Vallé Q. Roques B. B. Bousquet-Mélou A. Dahlhaus D. Ramon-Portugal F. Dupouy V.. (2021). Prediction of minocycline activity in the gut from a pig preclinical model using a pharmacokinetic-pharmacodynamic approach. Front. Microbiol. 12:671376. doi: 10.3389/fmicb.2021.671376, PMID: 34305836 PMC8299485

[ref28] Wistrand-Yuen E. Knopp M. Hjort K. Koskiniemi S. Berg O. G. Andersson D. I. (2018). Evolution of high-level resistance during low-level antibiotic exposure. Nat. Commun. 9:1599. doi: 10.1038/s41467-018-04059-1, PMID: 29686259 PMC5913237

